# Tissue factor pathway-related biomarkers in liver cancer: activated factor VII–antithrombin complex and tissue factor mRNA levels are associated with mortality

**DOI:** 10.1016/j.rpth.2023.102310

**Published:** 2024-01-02

**Authors:** Nicola Martinelli, Sara Moruzzi, Silvia Udali, Annalisa Castagna, Laura Di Santo, Francesca Ambrosani, Marcello Baroni, Patrizia Pattini, Francesca Pizzolo, Andrea Ruzzenente, Simone Conci, Matthieu Grusse, Tommaso Campagnaro, Patrick Van Dreden, Alfredo Guglielmi, Francesco Bernardi, Oliviero Olivieri, Simonetta Friso

**Affiliations:** 1 Department of Medicine, University of Verona, Verona, Italy; 2 Department of Life Sciences and Biotechnology, University of Ferrara, Ferrara, Italy; 3 Department of Surgery, University of Verona, Verona, Italy; 4 Clinical Research Department, Diagnostica Stago, Gennevilliers, France

**Keywords:** activated factor VII–antithrombin, cholangiocarcinoma, hepatocellular carcinoma, prognosis, tissue factor mRNA expression

## Abstract

**Background:**

Tissue factor (TF), the main initiator of the coagulation cascade, plays a role in cancer progression and prognosis. Activated factor VII–antithrombin complex (FVIIa-AT) is considered an indirect marker of TF exposure by reflecting TF-FVIIa interaction.

**Objectives:**

To assess the link between FVIIa-AT plasma levels, *TF* messenger RNA (mRNA) expression, and survival in cancer.

**Methods:**

TF pathway–related coagulation biomarkers were assessed in 136 patients with cancer (52 with hepatocellular carcinoma, 41 with cholangiocarcinoma, and 43 with colon cancer) undergoing surgical intervention with curative intent. *TF* mRNA expression analysis in neoplastic vs nonneoplastic liver tissues was evaluated in a subgroup of 91 patients with primary liver cancer.

**Results:**

FVIIa-AT levels were higher in patients with cancer than in 136 sex- and age-matched cancer-free controls. In patients with cancer, high levels of FVIIa-AT and total TF pathway inhibitor were associated with an increased mortality risk after adjustment for confounders, but only FVIIa-AT remained a predictor of mortality by including both FVIIa-AT and total TF pathway inhibitor in Cox regression (hazard ratio, 2.80; 95% CI, 1.23-6.39; the highest vs the lowest quartile). This association remained significant even after adjustment for extracellular vesicle–associated TF-dependent procoagulant activity. In the subgroup of patients with primary liver cancer, patients with high *TF* mRNA levels had an increased mortality risk compared with that for those with low *TF* mRNA levels (hazard ratio, 1.92; 95% CI, 1.03-3.57), and there was a consistent correlation among high FVIIa-AT levels, high *TF* mRNA levels, and increased risk of mortality.

**Conclusion:**

High FVIIa-AT levels may allow the identification of patients with cancer involving high TF expression and predict a higher mortality risk in liver cancer.

## Essentials

• Activated factor (F)VII–antithrombin (FVIIa-AT) complex is an indirect marker of tissue factor (TF) activity.
• FVIIa-AT plasma levels and *TF* messenger RNA (mRNA) levels in liver tissues were assessed for liver cancer.
• Subjects with high *TF* mRNA levels in cancer tissues had an increased risk of mortality.
• High FVIIa-AT levels were associated with both high *TF* mRNA levels and increased risk of mortality.

## 1 Introduction

The deep and complex link between neoplastic disease and coagulation has been known for over 150 years, starting from Armand Trousseau’s first historical observation of thrombotic complications in a yet undiagnosed cancer [1]. Cancer can induce bidirectional and reciprocal abnormalities and derangements of the hemostatic system, thereby fostering both thrombotic and hemorrhagic complications [2].

Among the various players in the hemostatic balance of cancer, the transmembrane glycoprotein tissue factor (TF) is usually considered the most known and well-characterized tumor procoagulant factor. TF is a high-affinity receptor/cofactor for factor VII/activated FVII (FVIIa) and one of the key elements in hemostasis, in the form of a TF-FVIIa complex—the primary physiological initiator of the coagulation cascade by activating FIX and FX [3,4]. TF is constitutively expressed by vascular smooth muscle cells, fibroblasts, and perivascular cells, forming a hemostatic barrier and leading to a rapid initiation of the coagulation cascade when a blood vessel is damaged [5]. Activated monocytes express TF, while there is some controversy about TF expression by endothelial cells *in vivo*. TF expression can be induced by several triggers, including exposure to inflammatory cytokines [6]. Malignant tumor cells also express TF, and they can even release TF-positive extracellular vesicles (EVs) into the bloodstream [7], thus fostering both localized and systemic procoagulant states [2,8,9]. Nevertheless, the role of TF in cancer goes beyond prothrombotic risk and has been involved in tumor growth, angiogenesis, spreading, and metastasis development independently of clotting mechanisms [10]. Noteworthy, the TF-FVIIa complex also activates cell signaling by the cleavage of a G protein–coupled receptor, such as protease-activated receptor 2, thereby promoting cell adhesion and migration, as well as expression of proangiogenic proteins [2,8,11,12]. Experimental evidence in cell cultures and animal models suggests that TF-FVIIa enhances tumor growth and that TF inhibition may have anticancer effects [13,14]. Finally, novel anticancer therapies using TF expressed on the cancer cell surfaces as a target to deliver cytotoxic drugs to the tumor are in advanced development and even clinically tested. Tisotumab vedotin, a TF-directed antibody–drug conjugate [15], has been recently approved for the treatment of cervical cancer in clinical practice [16,17].

According to these premises, it would be clinically useful to have reliable and easily detectable biomarkers of TF expression/activity in patients with cancer. However, it is not easy to measure TF directly in plasma. TF is either a transmembrane protein, which can be found on EVs in plasma, or a soluble molecule, produced by alternative splicing (alternatively spliced TF [asTF]), leading to the lack of transmembrane domain but with minimal procoagulant activity [18]. It is worthy to note that several studies addressed extrahemostatic roles for asTF, including cancer-promoting properties, like favoring tumor growth and metastasis spreading [[19], [20], [21], [22]]. Furthermore, full-length, transmembrane TF exists in 2 conformational forms, having either low (encrypted) or high (decrypted) procoagulant activity. TF can be evaluated by means of either antigen or activity assays, but such assays do not accurately measure TF levels in plasma [23].

Antithrombin (AT), along with TF pathway inhibitor (TFPI), acts as an inhibitor of the TF-FVIIa pathway. Both AT and TFPI form stable complexes with TF-bound FVIIa, but only the FVIIa-AT complex is released and accumulates in the plasma, which offers an opportunity for an affordable measure. Thus, the plasma levels of FVIIa-AT have been proposed to indirectly reflect the global TF-FVIIa interaction and then, speculatively, TF expression/activity. A high FVIIa-AT plasma concentration has been shown as a potential biomarker of prothrombotic diathesis, correlating with increased activated FX (FXa) level and thrombin generation [24,25]. Notably, a high FVIIa-AT plasma concentration has also been associated with an increased risk of mortality in cardiovascular cohorts [24,26].

The clinical significance of FVIIa-AT plasma concentrations in patients with cancer is unknown. The aims of the present study, performed in a cohort of subjects with liver and colon cancer undergoing surgical intervention with curative intent, were as follows: (i) to relate FVIIa-AT plasma levels with those of other hemostatic biomarkers in the TF-FVIIa pathway, ie, FVII antigen (FVII Ag), total and free TFPI, and EV-associated TF-dependent procoagulant activity; (ii) to assess the relationship with *TF* messenger RNA (mRNA) expression in cancer tissues; and (iii) to compare the potential predictive role of these coagulation biomarkers in patients with cancer.

## 2 Methods

### 2.1 Study subjects

This observational study had both case-control and longitudinal design. One hundred thirty-six patients with cancer and 136 sex- and age-matched cancer-free control subjects were enrolled. Patients with cancer were enrolled from those being referred to the Division of Hepatobiliary Surgery of the Verona University Hospital (Verona, Italy) for curative surgery intervention, and they had either primary liver cancer, ie, hepatocellular carcinoma (HCC, *n* = 52) or intrahepatic cholangiocarcinoma (CC, *n* = 41), or secondary liver cancer, ie, colon cancer with synchronous liver metastasis (colon cancer, *n* = 43). Notably, patients with advanced liver cirrhosis and a history of hepatitis B virus or hepatitis B virus infection were excluded. Detailed enrollment criteria were previously reported [27,28]. Briefly, surgical resectability criteria were as follows: i) preserved liver function, defined by class A Child-Pugh score; and ii) the absence of extrahepatic metastases. Neoadjuvant chemotherapy before surgery was performed in 27 (19.9%) patients, mainly subjects with colon cancer (*n* = 23) and a few with HCC (*n* = 1) and CC (*n* = 3). Only 4 patients had a history of previous venous thromboembolism. Seven patients were taking warfarin, but oral anticoagulants were stopped 1 week before surgery and replaced by therapy with enoxaparin or heparin calcium. No subject was taking direct oral anticoagulants.

The cancer-free subjects (*n* = 136) were selected from the framework of the Verona Heart Study (VHS), a regional survey aimed to look for new risk factors of coronary artery disease in subjects with angiographic documentation of their coronary vessels [24,29]. These controls were selected on the basis of the following: i) the absence of any evidence and/or suspicion of neoplastic disease at the time of enrollment and ii) the availability of data on FVIIa-AT plasma levels. No subject within the control group was taking any anticoagulant drugs. Details about the VHS cohort have been previously reported [24,29].

A detailed clinical history was recorded at the time of enrollment for all subjects in both study groups. The time frame for enrollment of the subjects ranged from April 2009 to April 2018.

Patients with cancer were followed until death or until December 31, 2018. The status of study subjects was determined by periodic evaluation by means of ambulatory or telephone survey. No patient was lost during the follow-up period. All subjects enrolled in the study were European White. Both study protocols conformed to the ethical guidelines of the 1975 Declaration of Helsinki and were approved by the Ethical Review Board of the University of Verona School of Medicine Hospital (Verona, Italy). Written informed consent was obtained from each subject after a detailed explanation of the studies.

### 2.2 Laboratory analysis

Samples of venous blood were drawn from each subject after an overnight fast. Routine laboratory tests were performed for both patients with cancer and control subjects; the parameters tested were complete blood count, fibrinogen, prothrombin time (PT), activated partial thromboplastin time (aPTT), albumin, total bilirubin, aspartate aminotransferase, alanine aminotransferase, alkaline phosphatase, gamma-glutamyl transpeptidase, creatinine, total cholesterol, low-density lipoprotein cholesterol, high-density lipoprotein cholesterol, triglycerides, and glucose, and the values of these were determined by standard methods.

### 2.3 FVIIa-AT, FVII Ag, and total and free TFPI assays

The concentrations of FVIIa-AT, FVII Ag, and total and free TFPI were measured in frozen citrate plasma samples that had never been thawed before this study. Venous blood samples were drawn from each subject in 0.109-M (ie, 3.2% [weight/volume]) trisodium citrate anticoagulant and centrifuged at 2500 × *g* for 15 minutes at room temperature, and the plasma was collected and stored at −80 °C for subsequent analyses. Plasma samples were thawed in a water bath at 37 °C for 5 minutes before the assays. Enzyme-linked immunosorbent assay tests were performed for the quantitative determination of FVIIa-AT complex (Asserachrom VIIa-AT), FVII Ag (Asserachrom VII:Ag), total biologically available level of TFPI, ie, free and lipoprotein bound TFPI forms, (Asserachrom Total TFPI), and free TFPI (Asserachrom Free TFPI kit). All the assays were purchased from Diagnostica Stago S.A.S. and were performed according to the manufacturer’s instructions. All testing was performed in duplicate. The intra-assay and inter-assay coefficients of variations were <10%.

### 2.4 EV-associated TF-dependent procoagulant activity

EVs were pelleted from 500 μL of platelet-free plasma by centrifugation at 24,000 × *g* for 1 hour at 4 °C and washed twice with Hepes buffer saline with bovine serum albumin. After being suspended in a washing solution, the pellet was centrifuged again for 1 hour at 24,000 × *g* before being resuspended in the buffer. The TF-dependent procoagulant activity of the EVs was determined using the CY-QUANTTM MV-TF Activity Kit (Diagnostica Stago). Briefly, samples were incubated with either anti-TF (blocking monoclonal antibody [mAb]) or negative control (nonblocking mAb) for 30 minutes at 37 °C. Next, 20 μL of Hepes buffer saline with bovine serum albumin containing a mixture of FVII and FX plus CaCl_2_ was added to each sample, and the mixture was incubated for 2 hours at 37 °C. FXa generation was stopped by the addition of 20 μL of EDTA buffer, and 80 μL of the chromogenic substrate of FXa was added. Finally, absorbance at 405 nm (TF-dependent FXa generation) was measured at intervals of 60 seconds over a period of 15 minutes. TF activity was calculated by reference to a standard curve generated using relipidated recombinant human TF. The TF-dependent FXa generation (femto Molar) was determined by subtracting the Vmax value independent of TF (amount of FXa generated in the presence of an inhibitory anti-TF mAb; clone SBTF-1, BioCytex) from the total Vmax value (amount of FXa generated in the presence of the negative control antibody).

### 2.5 TF mRNA expression analyses in liver tissue samples

*TF* mRNA expression analysis was performed by real-time reverse-transcription polymerase chain reaction (RT-PCR) in 51 cases of HCC and 40 cases of CC by comparing *TF* mRNA expression levels in neoplastic and homologous nonneoplastic liver tissues, ie, a sample of liver tissue obtained from a region far from the tumor mass and that is histologically tumor-free. The RNA samples were extracted with TRI Reagent (Thermo Fisher Scientific) following the manufacturer’s protocol and quantified by NanoDrop One instrument (Thermo Fisher Scientific) and Qubit fluorometer using the RNA Broad-Range Assay Kit (Thermo Fisher Scientific). Reverse transcription was performed using the SuperScript VILO complementary DNA Synthesis Kit (Thermo Fisher Scientific), and evaluation of mRNA expression was performed on the 7500 Real-Time PCR System (Applied Biosystems) using *TF* TaqMan assays (Hs01076029 m1 F3, Applied Biosystems by Thermo Fisher Scientific) and *GAPDH* as the housekeeping gene (Hs99999905_m1). Two PCR replicates per complementary DNA sample were performed. The neoplastic and nonneoplastic tissues of each patient were analyzed in the same PCR plate. Levels of total *TF* mRNA were measured [30], and the relative mRNA expression was defined as fold change according to the 2^−ΔΔCt^ method [31]. *TF* mRNA levels were considered to be significantly lower in neoplastic tissue than in homologous nonneoplastic tissue when the fold change was <1, while *TF* mRNA levels were considered to be significantly higher when the fold change was >1.

To evaluate the interindividual variations in *TF* mRNA levels, the ΔCt_N_ value in nonneoplastic tissue was calculated for each patient (ΔCt_N_ = Ct_TF_ − Ct_GAPDH_) and then compared to the mean ΔCt value in nonneoplastic tissue according to the formula −ΔΔCt_N_ = −(ΔCt_N_ − mean ΔCt_N_).

### 2.6 Statistical analysis

All the analyses were performed using the IBM SPSS 23 statistical software (IBM Inc). Continuous variables are expressed as mean ± SD. Continuous variables showing a non-Gaussian distribution (eg, FVIIa-AT and free and total TFPI) were log-transformed and are expressed as geometric means with 95% CIs. Continuous variables were tested by Student’s *t*-test or analysis of variance with polynomial contrasts for linear trend when appropriate. Correlations between continuous variables were evaluated by the Pearson correlation test. Categorical variables were analyzed using chi-squared test or chi-squared test for linear trend when appropriate.

Survival analyses of patients with cancer were performed by means of Kaplan–Meier curves and assessed by log-rank test. The whole cancer population was stratified according to the quartiles of FVIIa-AT, FVII Ag, and total and free TFPI plasma concentration. Hazard ratios (HRs) of mortality with 95% CI based on either FVIIa-AT or total TFPI levels were estimated by Cox regression analysis, with the lowest quartile considered the reference group. Different Cox regression models were performed by including potential confounding factors, such as sex, age, body mass index (BMI), smoking, type of cancer, albumin, creatinine, fibrinogen, PT, and aPTT. Subjects with missing data were excluded from regression models. A *P* value of <.05 was considered statistically significant.

## 3 Results

### 3.1 Clinical and biochemical characteristics of the study population

The clinical and biochemical characteristics of the 136 subjects with liver cancer (patients with cancer) and the 136 sex- and age-matched subjects without a history of cancer (cancer-free controls) are summarized in Table 1.

**Table 1:** Clinical and biochemical characteristics of patients with cancer and control subjects.

Clinical and biochemical characteristics	Cancer-free controls (*n* = 136)	Patients with cancer (*n* = 136)
Clinical characteristics		
Age (y)	67.4 ± 9.2	67.4 ± 9.7
Sex (% males)	67.6	66.9
Race (% European Whites)	100.0	100.0
BMI (kg/m^2^)	26.6 ± 3.91	26.7 ± 4.13
Smoking (%)	61.5	57.0
Biochemical characteristics		
Hematocrit (%)	40.4 ± 4.35	40.3 ± 5.06
Hemoglobin (g/dL)	13.4 ± 1.57	13.1 ± 1.75
Erythrocytes (10^12^/L)	4.46 ± 0.52	4.44 ± 0.59
MCV (fL)^a^	90.5 (89.2-91.8)	90.8 (89.6-92.0)
Platelets (10^9^/L)^a^	237 (224-250)	228 (212-244)
White blood cells (10^9^/L)^a^	7.24 (6.89-7.61)	6.69 (6.28-7.13)
Fibrinogen (mg/dL)^a^	405 (385-426)	358 (341-375)
PT^a^	0.99 (0.98-1.01)	1.09 (1.07-1.12)^b^
aPTT^a^	0.99 (0.97-1.01)	1.02 (0.99-1.05)^b^
Albumin (g/L)^a^	39.4 (38.6-40.1)	39.0 (37.9-40.1)
Total bilirubin (mg/dL)^a^	0.63 (0.59-0.67)	0.70 (0.63-0.77)
AST (U/L)^a^	27.5 (24.6-30.9)	39.6 (34.4-45.4)
ALT (U/L)^a^	25.6 (22.8-28.6)	39.0 (32.4-46.9)
ALP (U/L)^a^	80.9 (69.8-93.7)	97.8 (88.5-108.0)
γGT (U/L)^a^	33.9 (29.8-38.6)	73.3 (61.8-86.8)
Creatinine (μmol/L)^a^	89.0 (85.2-93.0)	77.0 (71.1-83.4)
Total cholesterol (mmol/L)	4.95 ± 1.11	4.46 ± 1.23
LDL cholesterol (mmol/L)	3.28 ± 0.90	3.06 ± 0.70
HDL cholesterol (mmol/L)^a^	1.15 (1.10-1.21)	1.08 (1.01-1.16)
Triglycerides (mmol/L)^a^	1.55 (1.44-1.67)	1.33 (1.23-1.44)
Glucose (mmol/L)^a^	5.80 (5.51-6.10)	5.95 (5.68-6.23)

ALP, alkaline phosphatase; ALT, alanine aminotransferase; aPTT, activated partial thromboplastin time; AST, aspartate aminotransferase; BMI, body mass index; γGT, gamma-glutamyl transpeptidase; HDL, high-density lipoprotein; LDL, low-density lipoprotein; MCV, mean corpuscular volume; PT, prothrombin time.

a Log-transformed variables are shown as geometric mean with 95% CI.

b Seven patients with cancer who underwent warfarin therapy were excluded.

The clinical and biochemical characteristics of patients with cancer according to the type of cancer (ie, HCC, CC, and metastasis of colon cancer) are reported in Supplementary Table S1. Patients with HCC were older, more represented by males, and with a higher alcohol intake than those affected by CC or colon cancer. They also had lower total cholesterol and fibrinogen levels and slightly longer PT (Supplementary Table S1).

### 3.2 FVIIa-AT, FVII Ag, total and free TFPI, and EV-TF-procoagulant activity assays in the study population and survival analysis

Although with a substantial overlap of values, FVIIa-AT plasma levels were higher in patients with cancer than in cancer-free controls (92.5 pM [95% CI, 86.4-99.0 pM] vs 82.6 pM [95% CI, 76.9-88.7 pM]; *P* = .024), as shown in Supplementary Figure S1. In the cancer group, FVII Ag and total and free TFPI plasma levels were also determined. All these parameters correlated directly with FVIIa-AT levels as well as reciprocally among each other (Supplementary Table S2). In contrast, no correlation was found for EV-associated TF-dependent procoagulant activity with any of the aforementioned 4 coagulation biomarkers (Supplementary Table S2). Stratifying the neoplastic cohort according to cancer type, patients with CC showed the highest levels of FVIIa-AT, FVII Ag, and total and free TFPI, while subjects with colon cancer had the lowest levels of EV-associated TF-dependent procoagulant activity (Supplementary Table S3). These results were also confirmed after excluding the 27 subjects who had previously received neoadjuvant chemotherapy before surgery (data not shown), which was not associated with different FVIIa-AT plasma levels (92.9 pM [95% CI, 86.0-100.4 pM] vs 90.7 pM [95% CI, 78.6-104.7 pM]; *P* = .782).

**Figure 1:**
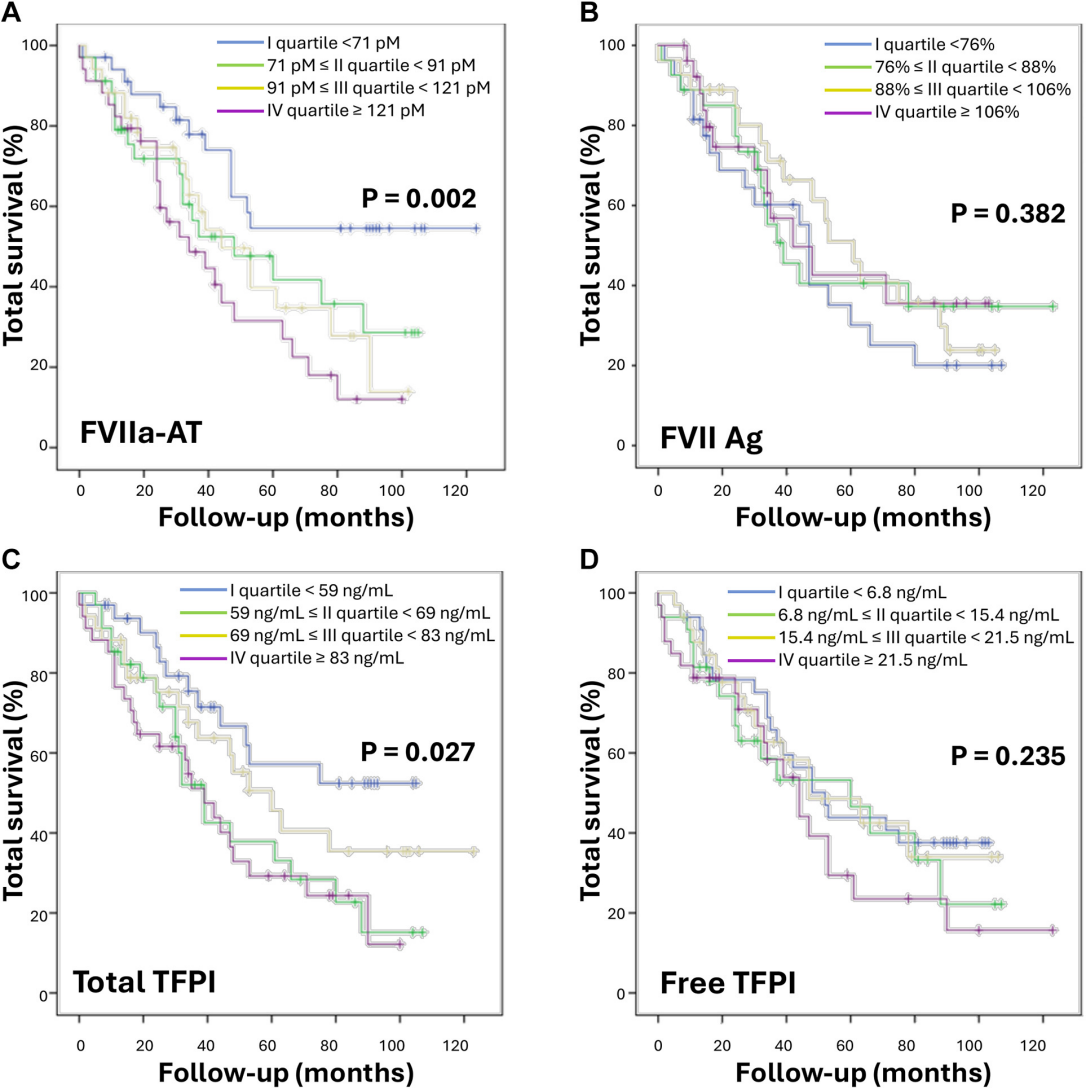
Kaplan–Meier survival curves related to coagulation factors. Survival curves are shown according to activated factor VII–antithrombin (FVIIa-AT) (A), factor VII antigen (FVII Ag) (B), total (C), and free tissue factor pathway inhibitor (TFPI) (D) plasma levels in the whole cancer cohort (*n* = 136). Survival analyses were assessed by log-rank test.

Survival analysis was performed according to the quartile distribution of FVIIa-AT, FVII Ag, and total and free TFPI plasma concentration (Figure 1). After a 34-month median follow-up, 74 out of 136 patients with cancer (54.4%) died. The clinical and laboratory characteristics of the study population stratified according to quartile distribution of FVIIa-AT plasma levels are reported in Supplementary Table S4. The Kaplan–Meier survival curves showed that the mortality rate increased from the lowest to the highest quartile significantly for either FVIIa-AT (38.2%→52.9%→55.9%→70.6%; *P* = .002) or total TFPI plasma levels (36.4%→61.8%→50.0%→70.6%; *P* = .027), while no association was found for FVII Ag and free TFPI (Figure 1). Moreover, EV-TF procoagulant activity was not associated with mortality rate (Supplementary Figure S2).

The strength of association of either FVIIa-AT or total TFPI levels with mortality was then assessed by means of multiple Cox regression models (Table 2). Subjects within the highest quartile of FVIIa-AT levels had approximately a 3-fold increased risk of mortality compared with those within the lowest quartile in univariate analysis (HR, 2.99; 95% CI, 1.51-5.91; *P* = .002). This mortality risk was confirmed by including sex, age, type of cancer, smoking habits, BMI, albumin, creatinine levels, traditional coagulation parameters (PT, aPTT, and fibrinogen), and total TFPI in regression models (Table 2). The association with mortality was confirmed even after adjustment for EV-TF-procoagulant activity (HR, 3.18; 95% CI, 1.59-6.34; *P* = .001). All these results were confirmed after exclusion of the 7 subjects taking warfarin, which was stopped 1 week before surgery (HR, 2.61; 95% CI, 1.30-5.22; *P* = .007); after exclusion of the 4 subjects who died during surgery (HR, 3.04; 95% CI, 1.49-6.19; *P* = .002); and after exclusion of the 27 subjects who had previously received neoadjuvant chemotherapy before surgery (HR, 3.12; 95% CI, 1.39-6.99; *P* = .006).

**Table 2:** Risk of mortality based on quartile distribution of activated factor VII–antithrombin or tissue factor pathway inhibitor plasma levels in different Cox regressions models (from unadjusted to multiadjusted) in the whole study population (*n* = 136).

Cox regression models	Risk of mortality for FVIIa-AT plasma levels				Risk of mortality for total TFPI plasma levels			
	FVIIa-AT quartiles				Total TFPI quartiles			
	I <71 pM *n* = 34 13 deaths	II 71-90.9 pM *n* = 34 18 deaths	III 91-120.9 pM *n* = 34 19 deaths	IV ≥121 pM *n* = 34 24 deaths	I <59 ng/mL *n* = 34 12 deaths	II 59-68.9 ng/mL *n* = 34 21 deaths	III 69-82.9 ng/mL *n* = 34 17 deaths	IV ≥83 ng/mL *n* = 34 24 deaths
Unadjusted	1	2.01 (0.98-4.12)	2.13 (1.05-4.34)	2.99 (1.51-5.91)	1	2.33 (1.14-4.75)	1.56 (0.74-3.26)	2.57 (1.28-5.16)
Model 1	1	1.94 (0.93-4.03)	2.11 (1.02-4.34)	2.94 (1.46-5.92)	1	2.29 (1.12-4.69)	1.47 (0.69-3.13)	2.45 (1.19-5.05)
Model 2	1	2.33 (1.04-5.22)	2.82 (1.26-6.33)	3.33 (1.54-7.20)	1	2.20 (1.02-4.75)	1.26 (0.52-3.06)	2.41 (1.05-5.56)
Model 3	1	2.45 (1.07-5.60)	2.92 (1.29-6.62)	3.54 (1.58-7.94)	1	2.60 (1.13-6.01)	1.27 (0.50-3.26)	2.44 (0.95-6.27)
Model 4	1	2.12 (0.91-4.94)	2.66 (1.15-6.18)	2.80 (1.23-6.39)	1	2.13 (0.88-5.16)	1.09 (0.41-2.92)	1.78 (0.67-4.75)

Subjects within the lowest quartile were considered as the reference group. Model 1: adjustment for sex and age. Model 2: adjustment for sex, age, type of cancer, smoking, body mass index, albumin, and creatinine. Model 3: adjustment for prothrombin time, activated partial thromboplastin time, and fibrinogen. Model 4: adjustment for prothrombin time, activated partial thromboplastin time, and fibrinogen, including both FVIIa-AT and total TFPI as independent variables in the Cox regression models.

FVIIa-AT, activated factor VII–antithrombin; TFPI, tissue factor pathway inhibitor.

The association between high FVIIa-AT plasma levels and increased mortality rate was found in the subgroup analysis of patients with primary liver carcinoma after excluding subjects with colon cancer (Table 3). Moreover, in patients with primary liver cancer (HCC and CC), the mortality risk was also confirmed after adjustment for microvascular invasion (HR, 3.02; 95% CI, 1.18-7.73; *P* = .021).

**Table 3:** Risk of mortality based on quartile distribution of activated factor VII–antithrombin or tissue factor pathway inhibitor plasma levels in different Cox regressions models (from unadjusted to multiadjusted) in the subgroup of subjects with primary liver cancer (hepatocellular carcinoma + cholangiocarcinoma, *n* = 93).

Cox regression models	Risk of mortality for FVIIa-AT plasma levels				Risk of mortality for total TFPI plasma levels			
	FVIIa-AT quartiles				Total TFPI quartiles			
	I <71 pM *n* = 24 8 deaths	II 71-90.9 pM *n* = 24 11 deaths	III 91-120.9 pM *n* = 23 10 deaths	IV ≥121 pM *n* = 22 13 deaths	I <59 ng/mL *n* = 26 8 deaths	II 59-68.9 ng/mL *n* = 24 12 deaths	III 69-82.9 ng/mL *n* = 20 8 deaths	IV ≥83 ng/mL *n* = 23 14 deaths
Unadjusted	1	2.06 (0.82-5.14)	1.86 (0.73-4.77)	2.67 (1.10-6.48)	1	2.19 (0.89-5.37)	1.89 (0.71-5.05)	2.59 (1.08-6.21)
Model 1	1	2.25 (0.89-5.69)	2.07 (0.79-5.40)	3.04 (1.21-7.63)	1	2.26 (0.92-5.58)	2.14 (0.78-5.89)	3.11 (1.22-7.95)
Model 2	1	2.96 (1.01-8.70)	3.05 (0.99-9.42)	3.57 (1.18-10.82)	1	1.93 (0.72-5.18)	1.90 (0.59-6.18)	3.37 (1.10-10.34)
Model 3	1	3.90 (1.23-12.35)	3.48 (1.09-11.06)	4.14 (1.32-12.97)	1	2.53 (0.85-7.54)	2.15 (0.64-7.20)	4.26 (1.26-14.40)
Model 4	1	3.62 (1.12-11.71)	2.69 (0.80-9.06)	3.96 (1.11-14.16)	1	1.72 (0.51-5.86)	2.38 (0.67-8.39)	3.54 (0.98-12.74)

Subjects within the lowest quartile were considered as the reference group. Model 1: adjustment for sex and age. Model 2: adjustment for sex, age, type of cancer, smoking, body mass index, albumin, and creatinine. Model 3: adjustment for prothrombin time, activated partial thromboplastin time, and fibrinogen. Model 4: adjustment for prothrombin time, activated partial thromboplastin time, and fibrinogen, including both FVIIa-AT and total TFPI as independent variables in the Cox regression models.

FVIIa-AT, activated factor VII–antithrombin; TFPI, tissue factor pathway inhibitor.

High levels of total TFPI were similarly associated with an increased risk of mortality in univariate analysis, with subjects within the highest quartile of total TFPI levels having a higher risk than those of subjects within the lowest quartile (HR, 2.57; 95% CI, 1.28-5.16; *P* = .008). Although the related risk of mortality was confirmed after adjustment for sex, age, type of cancer, smoking habits, BMI, albumin, and creatinine levels, the statistical significance was lost by including coagulation parameters and FVIIa-AT in the regression models (Tables 2 and 3).

### 3.3 *TF* mRNA expression and survival analysis in primary liver cancer

**Figure 2:**
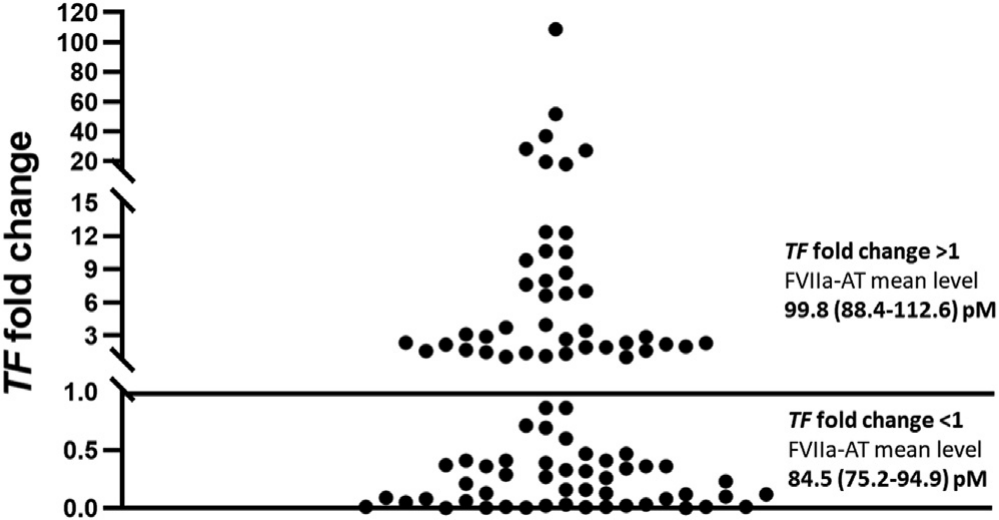
Distribution of tissue factor (*TF*) gene expression in subjects with primary liver cancer (*n* = 91). Activated factor VII–antithrombin (FVIIa-AT) complex mean plasma levels according to low (fold change, <1) or high *TF* messenger RNA levels (fold change, >1) are reported.

*TF* mRNA expression analysis of the *TF* gene in liver tissues was performed in a subgroup of patients with primary liver cancer (*n* = 91). There was a wide range of *TF* mRNA expression variability since 49 cancer tissues showed lower *TF* mRNA levels, while 42 cancer tissues had higher *TF* mRNA levels than homologous nonneoplastic liver tissue (Figure 2). Despite the large interindividual variability of *TF* mRNA levels observed in nonneoplastic tissues, even when considering HCC and CC separately, when −ΔΔCt_N_ values were compared between patients with high *TF* mRNA levels and patients with low *TF* mRNA levels in cancer tissue, the differences were not statistically significant (0.48 vs −0.41, respectively; *P* = .193 by *t*-test).

**Figure 3:**
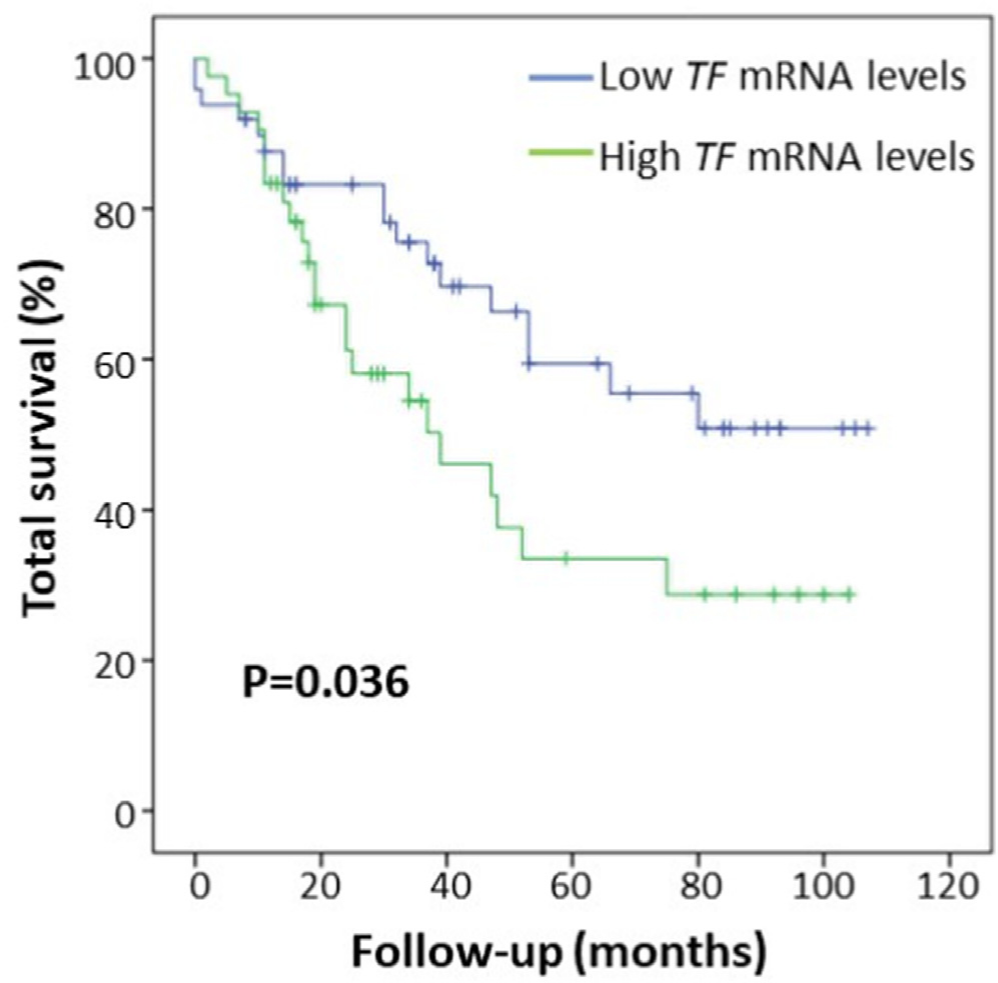
Tissue factor (TF) Kaplan–Meier survival curves. Curves indicate survival of patients with cancer according to *TF* gene expression in liver cancer tissues in subjects with primary liver cancer (*n* = 91). mRNA, messenger RNA.

Kaplan–Meier survival curves according to *TF* mRNA expression showed an increased mortality rate in patients with a TF fold change >1 compared to those with a TF fold change <1 (54.8% vs 36.7%; *P* = .036; Figure 3). High TF mRNA levels in cancer tissue were associated with an increased risk of mortality of about 2-fold (HR, 1.92; 95% CI, 1.03-3.57; *P* = .040) by the unadjusted Cox regression model. This finding was confirmed after adjustment for sex, age, and creatinine levels (HR, 1.98; 95% CI, 1.04-3.77; *P* = .038) and even after including microvascular invasion in the regression model (HR, 2.21; 95% CI, 1.09-4.46; *P* = .028).

### 3.4 High FVIIa-AT plasma levels are associated with both high *TF* mRNA levels and an increased risk of mortality in primary liver cancer

**Figure 4:**
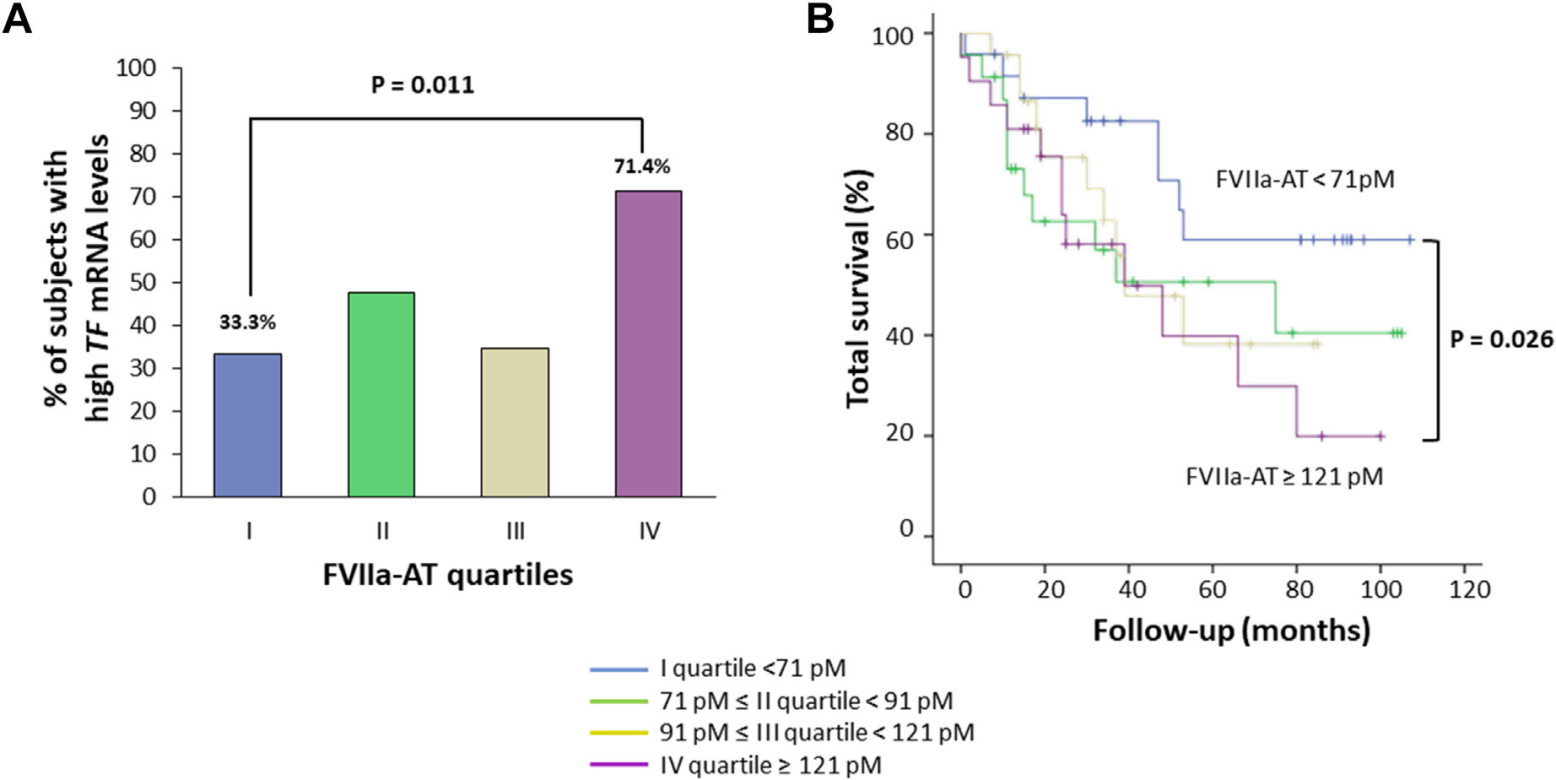
Tissue factor (TF) gene expression. (A) Prevalence of higher *TF* messenger RNA (mRNA) levels in cancer tissue and (B) Increased mortality rate by increasing activated factor VII–antithrombin (FVIIa-AT) plasma concentration, stratified in quartile distribution, in subjects with primary liver cancer (*n* = 91). *P* values by were calculated by (A) chi-squared test or (B) log-rank test.

Considering the subgroup of patients with primary liver cancer for whom data on *TF* mRNA expression analysis were available (*n* = 91), subjects with high *TF* mRNA levels in cancer tissue had marginally higher FVIIa-AT plasma levels than those in subjects with low *TF* mRNA levels (99.8 pM [95% CI, 88.4-112.6] vs 84.5 pM [95% CI, 75.2-94.9 pM]; *P* = .050). Stratifying this subsample according to FVIIa-AT plasma concentration, the proportion of subjects with high *TF* mRNA levels in cancer tissue increased progressively from the lowest to the highest quartile (*P* = .037 by chi-squared test for linear trend, Figure 4A). Finally, comparing subjects within the extreme FVIIa-AT quartiles, those within the highest quartile not only had an increased incidence of high *TF* mRNA levels (*P* = .011 by chi-squared test, Figure 4A) but also were confirmed to have an increased risk of mortality by Kaplan–Meier curves (*P* = .026 by log-rank test; Figure 4B).

## 4 Discussion

In the present study, we show that FVIIa-AT plasma levels in a small cohort of patients with cancer and *TF* mRNA expression in primary liver cancer may predict the overall survival in patients undergoing surgical intervention with curative intent. It is worthy to note that high FVIIa-AT plasma levels, high *TF* mRNA expression, and increased risk of mortality all appeared to be consistently associated, thus suggesting that FVIIa-AT may be a biomarker with both a functional and a prognostic significance.

The close relationship between cancer and TF pathway is well recognized even for functions that go beyond cancer-associated prothrombotic diathesis by modulating non-hemostatic processes and with ample evidence in several contests from mouse models [32,33] to neoplastic human diseases [13,34]. The great interest in the potential clinical relevance of TF assessment in patients with cancer is dampened by the difficulties related to TF assays that do not appear to accurately measure TF activity in plasma. TF, in fact, exists in different molecular forms (full-length protein with transmembrane localization, which can be isolated on EVs in plasma, and alternatively spliced soluble form) and in different conformational forms (encrypted or decrypted form of full-length TF). Such obstacles may be partially circumvented by measuring indirectly the global TF-FVIIa interaction through the FVIIa-AT assay.

The first finding of our analysis was the observation of higher FVIIa-AT concentrations in the plasma of patients affected by liver and colon cancer compared with those in the plasma of cancer-free controls. Considering the distribution of FVIIa-AT plasma levels in our study groups, the post hoc statistical power was 70.7% with an alpha of 0.05 [35], with the limitation that post hoc power analysis had been criticized and the related results could be misinterpreted [36]. No previous studies investigated the link between FVIIa-AT complex and cancer. Nonetheless, this result appears consistent with many earlier studies showing an increased expression of *TF* mRNA in various cancers, such as those affecting the colon rectum, pancreas, and breast [[37], [38], [39], [40], [41], [42]]. It should be noted that malignant cells are not the unique source of *TF* expression in patients with cancer. TF has been detected on the surface of plasma EVs derived from tumor cells in several types of malignancies, such as stomach, colorectal, pancreaticobiliary, lung, and breast cancers [[43], [44], [45], [46]]. Finally, *TF* expression can be induced on the cellular membranes of either endothelial cells (although there is some controversy about this) or monocytes by various triggers, including inflammatory cytokines and growth factors, which are well known to be crucial players in cancer pathophysiology [6,47]. Therefore, all these underlying molecular pathways may contribute to the increased *TF* expression and, consequently, to the TF-FVIIa interaction in cancer (Supplementary Figure S3). Although the issue of different sources of TF cannot be disentangled by a single laboratory plasma assay, FVIIa-AT can provide a surrogate marker of global *TF* expression. On the other hand, we cannot exclude that cancer cells may influence other molecular mechanisms modulating FVIIa-AT, including FVIIa [48] and AT [49]. We carried out this project to find proof supporting the idea that plasma levels of FVIIa-AT indirectly reflect *TF* expression in cancer tissue.

Although the molecular reasons indicating FVIIa-AT as a surrogate biomarker of TF-FVIIa interaction *in vitro* are reliable [50], evidence supporting this role *in vivo* is scarce. Few reports suggested that plasma levels of FVIIa-AT directly correlated with *TF* mRNA expression in leukemia cells [51,52]. Our data showed that patients with high *TF* mRNA levels in cancer tissues had higher FVIIa-AT plasma levels as well as a 2-fold increased risk of mortality compared with those in patients having low *TF* mRNA levels in cancer tissues. There was a progressive increase in the proportion of subjects with high *TF* mRNA levels by increasing FVIIa-AT plasma levels, thus suggesting that a high FVIIa-AT plasma concentration may allow the identification of subjects with highly *TF*-expressing cancers and a worse prognosis.

Earlier studies not only showed an increased TF expression/activity in malignancies but also demonstrated that such an increase was associated with a poor prognosis, even independently of thrombotic complications [[37], [38], [39], [40], [41], [42]]. Notably, some studies addressed the role of TF specifically in primary liver cancer: i) *TF* expression was correlated with tumor angiogenesis in HCC tissue samples, while increased TF levels were associated with decreased survival [53]; ii) *TF* expression was an independent risk factor of recurrence in patients undergoing surgery of curative hepatectomy for HCC [54]; and iii) TF levels were higher in both plasma and neoplastic tissues of patients with HCC and were associated with the index of invasion and metastasis [55].

Consistently with these observations, our results showed that a single assessment of FVIIa-AT plasma concentration before surgery with curative intent predicted the risk of mortality in patients with primary liver and colon cancer. The association with mortality was confirmed after adjustment for multiple confounders, including total TFPI, a key biomarker of the TF pathway whose plasma concentration directly correlated with FVIIa-AT levels and was associated with mortality rates in univariate analysis (but not after adjustment for FVIIa-AT levels). Bearing in mind the antithrombotic role of TFPI, the direct association of TFPI levels and mortality rate may appear counterintuitive at first glance, but high TFPI levels have also been reported as markers of endothelial cell dysfunction [56]. Unfortunately, no other biomarkers of endothelial cell activation were available in this study population, allowing us to further explore this hypothesis. Nevertheless, the association between TFPI levels and survival was lost after adjustment for FVIIa-AT levels. Finally, taking into account that EVs have been frequently claimed as risk factors and prognostic predictors in cancer [57], it is worthy to note that the association of FVIIa-AT plasma concentration and mortality was also confirmed after adjustment for EV-TF-procoagulant activity, another biomarker of TF pathway specifically related to EVs. Remarkably, in the multivariable modeling, the association between high FVIIa-AT levels and mortality was not only significant but remained unchanged and unattenuated with approximately a 3-fold increase in risk.

The result for risk of mortality among patients with cancer reiterates the findings obtained within cardiovascular cohorts. In a previous study, within the frame of the angiographically controlled VHS, we showed that high plasma levels of FVIIa-AT predict total and cardiovascular mortality in patients with stable coronary artery disease [24]. This observation was confirmed in the cardiovascular cohort of the Cardiovascular Health Study, where high FVIIa-AT plasma concentration was associated with an increased risk of overall mortality [26]. All these results seem to indicate that an increased TF-FVIIa interaction, marked by high FVIIa-AT levels, may be a common molecular mechanism underlying unfavorable outcomes in different clinical settings, from cardiovascular disease to cancer.

To the best of our knowledge, this is the first and only study associating FVIIa-AT plasma levels, *TF* mRNA expression in cancer tissues, and survival in a cohort of patients with liver cancer. Our findings suggest that the FVIIa-AT complex, an easily detectable plasma biomarker, may allow the identification of patients with cancer characterized by an enhanced neoplastic expression of *TF* and a consequent greater risk of mortality. We are tempted to speculate that patients with cancer with higher FVIIa-AT levels, marking increased *TF* mRNA levels, may have the greater benefit of TF-tailored therapies, from FXa inhibitors to TF-directed antibody–drug conjugates. For the former therapies, it is worthy to note that recent proofs suggest that the net benefit of FXa inhibitors in cancer may be at least in part independent of their antithrombotic effects, for instance, by enhancing the efficacy of immune checkpoint inhibitors [58], which in turn may stimulate an increased *TF* expression [59]. For the latter therapies, such an approach appears as a new tool for a molecularly targeted treatment against malignant cells, similar to the case of tisotumab vedotin, recently approved for cervical cancer, which could be the forerunner of a new class of anticancer anti-TF agents [15,16].

Our study has some limitations that should be acknowledged: the small and heterogeneous study sample and the lack of a second population for replication analysis. It should be noted that in the case-control analysis, patients with cancer were compared to subjects without cancer but with cardiovascular disease (ie, not healthy individuals). Gene expression analyses in liver tissue samples were performed for *TF*, but not potentially interesting coagulation players, like FVIIa, AT, or thrombomodulin. The calculation of *TF* fold change for each patient considering the homologous nonneoplastic tissue allowed us to consider the interindividual variability determining the differences in *TF* mRNA levels between neoplastic and nonneoplastic tissues but did not allow us to quantify the absolute mRNA levels. Furthermore, no protein-level data on TF expression in cancer tissues were available. Also, the laboratory assessment was relatively limited: no data on other biomarkers of either coagulation activation (such as thrombin–AT complex and FXa levels) or endothelial cell activation were available, and data on asTF were lacking. Regarding coagulation biomarkers, in earlier works in cardiovascular cohorts, we showed that high FVIIa-AT were associated with increased FXa and thrombin generation, thereby suggesting FVIIa-AT as an indicator of hypercoagulability [24,25] and, noteworthy, both FXa and thrombin generation have been related with a worse prognosis in cancer [60,61]. Nonetheless, FVIIa-AT results remained significant after adjustment for traditional coagulation parameters as well as after adjustment for total TFPI and EV-TF-procoagulant activity, among several quantified biomarkers of the TF pathway. The lack of important clinical data during follow-up, such as thrombotic complications and cause of death, should also be emphasized, but the main result for overall survival remains impressive in supporting the potential prognostic role of FVIIa-AT plasma concentration assessment in patients with cancer.

In summary, in this study, we provide the first clinical evidence consistently linking FVIIa-AT plasma levels, *TF* mRNA expression levels in malignant tissues, and overall survival in patients with liver cancer. Certainly, it is premature to claim that FVIIa-AT levels can be used to estimate TF expression and activity in cohorts of neoplastic patients, and our results need to be validated in further larger studies with a prospective design. Nonetheless, our data address the role of the TF pathway in cancer and may pave the way to new treatment strategies in the management of patients with cancer.
