# Supplementary material for: Tissue factor pathway-related biomarkers in liver cancer: activated factor VII–antithrombin complex and tissue factor mRNA levels are associated with mortality
Source: Res Pract Thromb Haemost. 2024 Jan 2;8(1):102310. doi: 10.1016/j.rpth.2023.102310 (PMC10818084; doi:10.1016/j.rpth.2023.102310)
Supplement: Supplementary material [file mmc1.doc]

**SUPPORTING MATERIALS**

**SUPPLEMENTARY TABLES**

**Supplementary Table 1. Clinical and biochemical characteristics of patients according to cancer type.**

|  |  | | **Hepatocellular carcinoma** (n=52) | **Cholangio-carcinoma** (n=41) | **Colon cancer**  (n=43) | | **P value** | |
| --- | --- | --- | --- | --- | --- | --- | --- | --- |
| **Clinical characteristics** |  | |  |  |  | |  | |
| Age (years) |  | | 70.0 ± 7.7 | 65.8 ± 10.7 | 65.7 ± 10.5 | | 0.047 | |
| Gender (% males) |  | | 86.5 | 48.8 | 60.5 | | <0.0001^§^ | |
| BMI (kg/m^2^) |  | | 27.6 ± 4.1 | 25.8 ± 3.9 | 26.4 ± 4.3 | | N.S. | |
| Smoking (%) | No | | 36.0 | 52.8 | 42.9 | |  | |
|  | Current | | 8.0 | 11.1 | 14.3 | | N.S.^§^ | |
|  | Previous | | 56.0 | 36.1 | 42.9 | |  | |
| Alcohol drinking (%) | No | | 20.0 | 58.3 | 50.0 | |  | |
|  | Current | | 52.0 | 36.1 | 45.2 | | <0.0001^§^ | |
|  | Previous | | 28.0 | 5.6 | 4.8 | |  | |
| **Biochemical characteristics** | | |  |  |  | |  | |
| Fibrinogen (mg/dL)* | |  | 322  (298 - 348) | 376  (343 - 413) | | 389  (361 - 420) | | 0.002 |
| PT* | |  | 1.13  (1.09– 1.17) | 1.09  (1.02 – 1.17) | | 1.06  (1.03 – 1.09) | | 0.028 |
| aPTT* | |  | 1.06  (1.01 – 1.12) | 0.99  (0.94 – 1.04) | | 1.00  (0.97 – 1.03) | | N.S. |
| Albumin (g/L)* | |  | 39.0  (37.5 - 40.6) | 39.3  (37.0 - 41.6) | | 38.5  (36.5 - 40.7) | | N.S. |
| Total bilirubin (mg/dL)* | |  | 0.81  (0.70 - 0.94) | 0.68  (0.56 - 0.82) | | 0.59  (0.50 - 0.71) | | 0.030 |
| AST (U/L)* | |  | 40.5  (32.3 - 50.7) | 35.2  (27.1 - 45.7) | | 41.9  (32.6 - 53.9) | | N.S. |
| ALT (U/L)* | |  | 41.8  (31.8 - 55.0) | 42.1  (27.7 - 63.8) | | 35.2  (25.5 - 48.6) | | N.S. |
| ALP (U/L)* | |  | 86.7  (75.8 - 99.1) | 112.1  (90.8 - 38.4) | | 102.3  (84.2 - 124.2) | | N.S. |
| GGT (U/L)* | |  | 74.6  (58.4 - 95.4) | 77.4  (55.4 -108.2) | | 68.5  (48.8 - 96.0) | | N.S. |
| Creatinine (µmol/L)* | |  | 78.2  (69.3 - 88.2) | 81.2  (65.6-100.4) | | 73.5  (67.4-82.0) | | N.S. |
| Total cholesterol (mmol/L) | |  | 3.99 ± 0.99 | 4.48 ± 1.33 | | 4.98 ± 1.24 | | 0.001 |
| LDL cholesterol (mmol/L) | |  | 2.76 ± 0.50 | 2.97 ± 0.62 | | 3.38 ± 0.79 | | 0.008 |
| HDL cholesterol mmol/L)* | |  | 1.06  (0.96 - 1.16) | 1.12  (0.95 - 1.33) | | 1.08  (0.95 - 1.23) | | N.S. |
| Triglycerides (mmol/L)* | |  | 1.21  (1.07 - 1.36) | 1.35  (1.15 - 1.58) | | 1.49  (1.30 - 1.70) | | N.S. |
| Glucose (mmol/L)* | |  | 6.46  (5.98 - 6.98) | 5.78  (5.30 - 6.30) | | 5.56  (5.15 - 5.99) | | 0.017 |

ANOVA test for evaluating mean value differences among the three cancer types.

^§^ χ^2^ test for categorical variables.

* log-transformed variables are shown as geometric mean with 95% confidence interval.

N.S. not statistically significant.

**Supplementary Table 2. Pearson’s correlation analysis among FVIIa-AT, FVII Ag, free and total TFPI plasma levels, and extracellular vesicles-associated TF-dependent procoagulant activity (EV-TF-procoagulant activity).**

| **Pearson's correlation parameters** | | **FVIIa-AT*** | **FVII Ag** | **Total**  **TFPI*** | **Free**  **TFPI*** | **EV- TF- procoagulant activity*** |
| --- | --- | --- | --- | --- | --- | --- |
| **FVIIa-AT*** | r |  | 0.375 | 0.350 | 0.329 | 0.028 |
|  | P |  | <0.0001 | <0.0001 | <0.0001 | 0.792 |
| **FVII Ag** | r | 0.375 |  | 0.303 | -0.261 | 0.031 |
|  | P | <0.0001 |  | 0.001 | 0.007 | 0.803 |
| **Total TFPI*** | r | 0.350 | 0.303 |  | 0.344 | 0.122 |
|  | P | <0.0001 | 0.001 |  | <0.0001 | 0.247 |
| **Free TFPI*** | r | 0.329 | -0.261 | 0.344 |  | 0.123 |
|  | P | <0.0001 | 0.007 | <0.0001 |  | 0.244 |
| **EV- TF- procoagulant activity*** | r | 0.028 | 0.031 | 0.122 | 0.123 |  |
|  | P | 0.792 | 0.803 | 0.247 | 0.244 |  |

*Log-transformed variables.

**Supplementary Table 3. Plasma levels of FVIIa-AT, FVII Ag, Total TFPI and free-TFPI, and extracellular vesicles-associated TF-dependent procoagulant activity (EV-TF-procoagulant activity) in cancer patients according to cancer type.**

|  | **Hepatocellular carcinoma**  (n=52) | **Cholangio-carcinoma**  (n=41) | **Colon cancer**  (n=43) | **P value** # |
| --- | --- | --- | --- | --- |
| **FVIIa-AT (pM)*** | 81.4 (73.0-90.7) | 105.9 (94.3-119.1) | 94.8 (83.9-107.1) | 0.005 |
| **FVII Ag (%)** | 85.9 ± 22.6 | 98.9 ± 21.5 | 89.3 ± 21.3 | 0.048 |
| **Total TFPI (ng/mL)*** | 62.3 (57.1-67.9) | 75.9 (70.0-82.2) | 71.7 (66.2-77.6) | 0.002 |
| **Free TFPI (ng/mL)*** | 11.0 (9.26-13.1) | 16.0 (13.9-18.6) | 12.7 (10.3-15.7) | 0.012 |
| **EV- TF- procoagulant activity (fM)*** | 4.92 (3.22-7.52) | 5.71 (3.21-9.63) | 2.46 (1.50-4.02) | 0.043 |

*Log-transformed variables are shown as geometric mean with 95% CI.

#by ANOVA

**Supplementary Table 4. Clinical and laboratory characteristics of the study population stratified according to quartile distribution of FVIIa-AT plasma levels.**

|  |  | **FVIIa-AT plasma levels (pM)** | | | | **P value** |
| --- | --- | --- | --- | --- | --- | --- |
|  |  | **<70.7** | **70.7-90.6** | **90.6-121.5** | **>121.5** |  |
| **Clinical Characteristics** |  |  |  |  |  |  |
| Age (years) |  | 64.3±9.2 | 66.8±10.5 | 68.3±9.0 | 70.2±9.7 | 0.010 |
| Gender (% males) |  | 73.5 | 64.7 | 61.8 | 67.6 | N.S. |
| BMI (kg/m^2^) |  | 28.4±3.9 | 26.1±3.4 | 26.8±5.0 | 25.6±3.7 | 0.020 |
| Smoking (%) | No | 38.2 | 56.7 | 42.4 | 35.5 |  |
|  | Current | 0.0 | 3.3 | 15.2 | 25.8 | 0.011 |
|  | Previous | 61.8 | 40.0 | 42.4 | 38.7 |  |
| Alcohol drinking (%) | No | 44.1 | 50.0 | 33.3 | 35.5 |  |
|  | Current | 35.3 | 40.0 | 51.5 | 54.8 | N.S. |
|  | Previous | 20.6 | 10.0 | 15.2 | 9.7 |  |
| **Biochemical characteristics** | |  |  |  |  |  |
| Fibrinogen (mg/dL)* |  | 318  (285-355) | 361  (333-392) | 373  (341-409) | 384  (347-425) | 0.006 |
| PT* |  | 1.14  (1.12-1.25) | 1.07  (1.03-1.10) | 1.07  (1.03-1.11) | 1.06  (0.99-1.13) | 0.002 |
| aPTT* |  | 1.07  (1.00-1.14) | 1.03  (0.96-1.10) | 0.98  (0.94-1.01) | 1.01  (0.95-1.07) | N.S. |
| Albumin (g/L)* |  | 36.5  (34.3-38.7) | 38.7  (36.4-41.2) | 41.5  (39.7-43.4) | 39.5  (37.4-41.7) | 0.011 |
| Creatinine (µmol/L)* |  | 66.4  (60.4-73.0) | 79.3  (67.3-93.4) | 78.9  (69.1-90.2) | 85.5  (67.8-107.8) | 0.035 |
| Total cholesterol (mmol/L) |  | 4.28±1.18 | 4.57±1.14 | 4.56±1.25 | 4.42±1.42 | N.S. |
| Triglycerides (mmol/L)* |  | 1.39  (1.18-1.63) | 1.28  (1.13-1.44) | 1.27  (1.05-1.54) | 1.41  (1.19-1.66) | N.S. |

ANOVA test with polynomial contrasts for linear trend for evaluating mean value differences.

^§^ χ^2^ test for linear trend for categorical variables.

* log-transformed variables are shown as geometric mean with 95% confidence interval.

N.S. not statistically significant.

Abbreviations: BMI, Body Mass Index; PT, Prothrombin Time; aPTT, activated Partial Thromboplastin Time

**SUPPLEMENTARY FIGURES**

**SUPPLEMENTARY FIGURE 1**


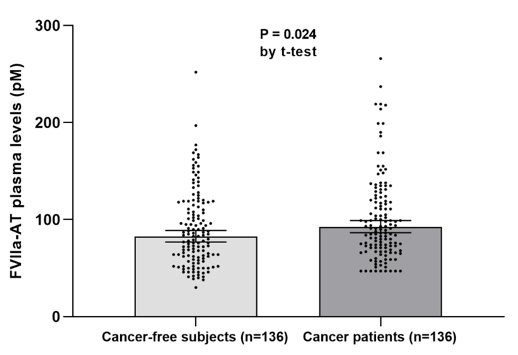


**Supplementary Figure 1.** FVIIa-AT plasma concentration in liver cancer cohort (n=136) compared with sex- and age-matched cancer-free controls (n=136)

**SUPPLEMENTARY FIGURE 2**


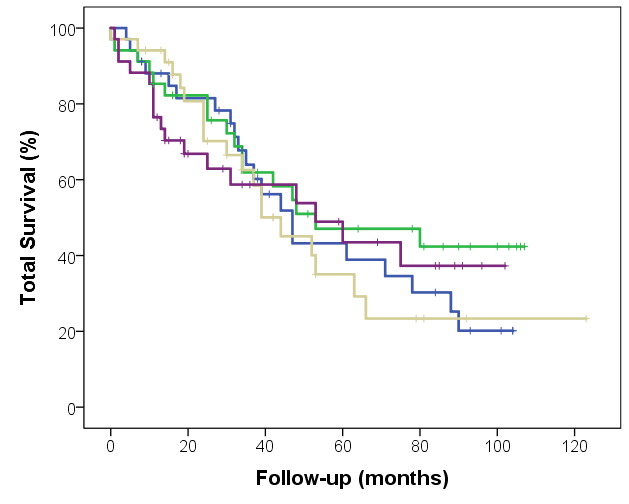


I quartile < 2.58 fM

2.58 ≤ II quartile < 4.82 fM

4.82 ≤ III quartile < 8.98 fM

IV quartile ≥ 8.98 fM

**P=0.871**

**EV-TF-procoagulant activity**

**Supplementary Figure 2.** Kaplan-Meier survival curves related to extracellular vesicles-associated TF-dependent procoagulant activity (EV-TF-procoagulant activity) in the whole cancer cohort (n=136). Survival analyses were assessed by Log-rank test.

**SUPPLEMENTARY FIGURE 3**

**Supplementary Figure 3.** Molecular pathways contributing to the increased tissue factor (TF) expression in cancer.

1. Shigemori C, Wada H, Matsumoto K, Shiku H, Nakamura S, Suzuki H. Tissue factor expression and metastatic potential of colorectal cancer. *Thromb Haemost*. 1998;80(6):894-898.
2. Seto S, Onodera H, Kaido T, et al. Tissue factor expression in human colorectal carcinoma: correlation with hepatic metastasis and impact on prognosis. *Cancer*. 2000;88(2):295-301.
3. Kobayashi S, Koizume S, Takahashi T, et al. Tissue factor and its procoagulant activity on cancer-associated thromboembolism in pancreatic cancer. *Cancer Sci*. 2021;112(11):4679-4691.
4. Nitori N, Ino Y, Nakanishi Y, et al. Prognostic significance of tissue factor in pancreatic ductal adenocarcinoma. *Clin Cancer Res*. 2005;11(7):2531-2539.
5. Khorana AA, Ahrendt SA, Ryan CK, et al. Tissue factor expression, angiogenesis, and thrombosis in pancreatic cancer. *Clin Cancer Res*. 2007;13(10):2870-2875.
6. Ueno T, Toi M, Koike M, Nakamura S, Tominaga T. Tissue factor expression in breast cancer tissues: its correlation with prognosis and plasma concentration. *Br J Cancer*. 2000;83(2):164-170.
7. 7. Hernandez C, Orbe J, Roncal C, et al. Tissue factor expressed by microparticles is associated with mortality but not with thrombosis in cancer patients. *Thromb Haemost*. 2013;110(3):598-608.
8. 8. Bharthuar A, Khorana AA, Hutson A, et al. Circulating microparticle tissue factor, thromboembolism and survival in pancreaticobiliary cancers. *Thromb Res*. 2013;132(2):180-184.
9. 9. Steffel J, Luscher TF, Tanner FC. Tissue factor in cardiovascular diseases: molecular mechanisms and clinical implications. *Circulation*. 2006;113(5):722-731.
10. 10. Witkowski M, Landmesser U, Rauch U. Tissue factor as a link between inflammation and coagulation. *Trends Cardiovasc Med*. 2016;26(4):297-303.
